# Development of a Fully Implantable Stimulator for Deep Brain Stimulation in Mice

**DOI:** 10.3389/fnins.2020.00726

**Published:** 2020-07-21

**Authors:** Michael Fleischer, Heinz Endres, Michael Sendtner, Jens Volkmann

**Affiliations:** ^1^Department of Neurology, University Hospital Würzburg, Würzburg, Germany; ^2^Department of Neurology, Essen University Hospital, Essen, Germany; ^3^University of Applied Science Würzburg-Schweinfurt, Schweinfurt, Germany; ^4^Institute of Clinical Neurobiology, University Hospital Würzburg, Würzburg, Germany

**Keywords:** deep brain stimulation, rodent model, neuroscience method, implantable stimulator, behavior (rodent)

## Abstract

**Introduction:**

Deep brain stimulation is an established method for the treatment of neurological and psychiatric disorders. To elicit the underlying mechanisms and explore new stimulation targets, rodent models are necessary. Cable bound external stimulation or portable devices limit movement of the animals and influence behavioral experiments. Therefore, implantable, individually programmed devices are required.

**Experimental procedure:**

The stimulator consists of an 8bit-microcontroller mounted on a square electrical board (10 × 10 mm). External control is enabled by a magnetic reed contact, as running control serves a white LED, running modes are displayed by flash codes. Stimulation parameters could be programmed in the range of pulse width: 60–500 μs, amplitude: up to 300 μA and frequency: 10–500 Hz. Power is supplied by two standard batteries. The device was implanted in 8–10 weeks old BALBc-mice. Functionality was examined by electrical stimulation of nucleus accumbens area with standard parameters for mice and determination of c-fos levels *in vitro* in brain slices.

**Results:**

The implanted microstimulators were well-tolerated by the mice, without impairment of free movement. Coating, external control, and monitoring of function with LED flash code proved to be fully adequate. Stimulation with standard stimulating parameters of nucleus accumbens elicited strong c-fos elevation on simulation site.

**Conclusion:**

We present a fully implantable stimulator for freely moving mice that meets the urgent need for further research on the effects of deep brain stimulation in rodent models. It offers the possibility to conduct behavioral experiments for up to 30 days of stimulation.

## Introduction

First introduced for treatment of Parkinson’s disease, DBS has also been applied to other types of movement disorders such as dystonia (Mentzel et al., 2012), essential tremor (Deuschl et al., 2011) but also to refractory epilepsy (Fisher et al., 2010; Tykocki et al., 2012) and many other neuropsychiatric disorders (Cleary et al., 2015). It is increasingly used in the psychiatric field for obsessive-compulsive disorder (de Koning et al., 2011), treatment resistance depression (Taghva et al., 2012), addiction (Muller et al., 2013), Alzheimer’s disease (Hardenacke et al., 2012), Tourette syndrome (Viswanathan et al., 2012), and post-traumatic stress disorder (Novakovic et al., 2011). Thus, DBS offers new therapeutic approaches and is currently tested in a variety of clinical studies.

Although many patients are profiting from the effects of electrical stimulation and new fields of applications are accessed, the mechanisms behind the therapeutic effects are still not fully understood. In movement disorders, there are well-characterized animal models such as the MPTP model or the 6-OHDA model of Parkinson’s disease (Blesa and Przedborski, 2014), dystonia models (Wilson and Hess, 2013), as well as other models that are currently in use to investigate functional results in other diseases such as depressive-compulsive spectrum disorders (Camilla d’Angelo et al., 2014; Kocabicak et al., 2015) or fear and anxiety disorders (Reznikov et al., 2016). From animal models, the rational was drawn for STN stimulation in PD (Faggiani and Benazzouz, 2016). Interestingly, many studies on DBS in psychiatric disorders were first conducted in humans as for treatment-resistant depression (Mayberg et al., 2005). This has led to a situation that the clinical effects of DBS in such disorders are well-described, but the mechanisms of how DBS functions in these diseases are largely unknown. Rodent models have been established for depression (Nollet et al., 2013), compulsive-like behavior (Albelda and Joel, 2012), and drug addiction (Belin and Deroche-Gamonet, 2012; Hamani and Temel, 2012) which could provide valuable insights to understand the mechanisms how DBS works in these disorders and to optimize parameters and stimulation sites. However, the stimulators that are currently in use for rodents differ from those stimulators used in patients. Importantly, they normally are cable-bound and do not allow free movements. However, for behavioral analyses in rodents, methods are necessary allowing animals to move freely and to remain undisturbed over extended observation periods. Such methods for long-term stimulation are necessary so that they do not intervene with the study design by restricting free movements. Furthermore, despite that fact that functional outcomes are well-documented in animal models under DBS treatment, behavioral side effects which are needed to judge psychiatric side effects and other long-term behavioral effects of high-frequency stimulation still need more research. Thus, optimized stimulation methods are required to conduct such experiments.

Several possibilities exist for such experimental stimulation set-ups in animal models. So far, cable bound external stimulation is most commonly used. The implanted electrodes are fixed on the skull and externally connected with the stimulating device. The cable is connected via a swivel, which allows the animal to move around during the experiment while being connected. The field of the investigation is mostly narrowed to the size of a cage. Furthermore, the weight of the cable and the effect of limited movement which does not allow rodents to enter holding places should not be underestimated when investigating behavior in rats or mice. Portable devices help to overcome the spatial boundaries and depict a good alternative for cable bound stimulation (Forni et al., 2012). Also, for mice, such devices have been developed (de Haas et al., 2012). However, the relative weight of such devices also could be an obstacle for free movements in enriched environments that are commonly used for behavioral testing (Huttenrauch et al., 2016).

Here we introduce a fully implantable stimulator for rat and mouse models, which is freely programmable and has a battery running time of 30 days with DBS typical stimulating parameters. Pulses are delivered monophasic in constant current mode. This is specially designed for freely moving mice in behavioral tests to assess behavior that could help to understand potential side effects of DBS in neurological diseases and explore the possibilities of electrical stimulation in models of psychiatric illness. To test this new device, electrodes are implanted in nucleus accumbens and connected to the implanted stimulator. The *in vivo* stimulation showed robust induction of neuronal c-fos expression in stimulated brain areas, indicating that this device could be used for testing functional, behavioral, and also cellular effects of DBS in mouse models of neurological and psychiatric disorders over prolonged periods up to at least 30 days.

## Materials and Methods

### Experimental Procedure

**FIGURE 1:**
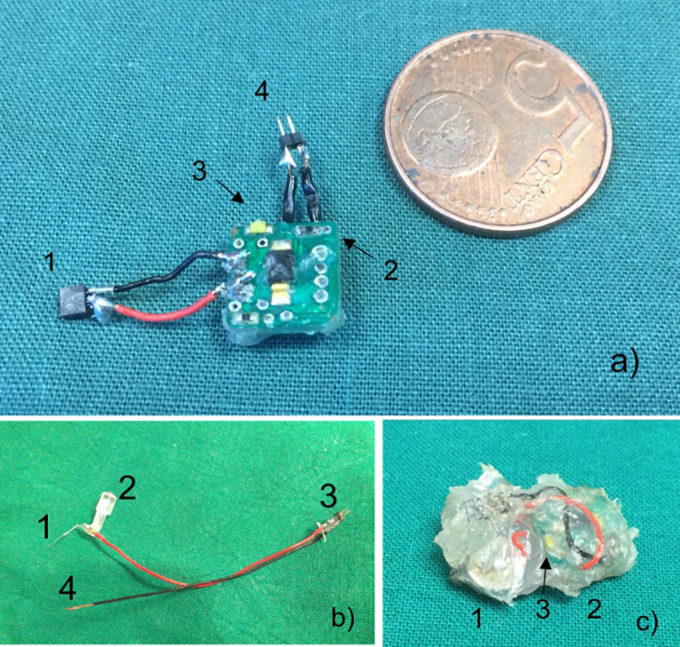
**(a)** Stimulator in comparison to 5 eurocent mint. Connection for electrode (1), magnetic reed contact (2), white LED for running control (3), connection to batteries (4). **(b)** Stimulation electrode with electrode (1), removable positioning aid (2), male connector (3), and connection to ground (4). **(c)** Coated stimulator device comprised of batteries (1), circuit board (2), and white LED (3).

The stimulator device consists of an implantable stimulator which is connected with the stimulation electrode. The board is equipped with a reed contact for switching the device magnetically “on” and “off” and a white high-power LED to read out the operational mode (Figure 1a). The light is visible through the skin. The board is a square with a length of the edge of 10 mm and a height of 1 mm. All edges are smoothed. Before implantation, the device was grouted with biocompatible silicon mass (Loctite^®^ 5248Tm, Henkel Technology) and cleaned in a bath containing 70% ethanol. The coating mass has an elastic texture, and special care was taken that all edges were coated thoroughly (Figure 1c).

### Electrodes

As electrodes commercially available, Teflon insulated platinum (90%)/iridium (10%) wire (Science Products GmbH, Hofheim) with 100 μm isolated and 50 μm uninsulated diameter was used in all experiments. The wire was connected to thin cables with an outer diameter of 1.2 mm and insulated with acrylic glue (Figure 1b). The stimulation electrode was stereotactically implanted using a positioning aid fixed by superglue to the acrylic insulation. After fixation of the electrode with dental cement, the positioning aid was removed. A screw which was positioned above the dura where the ground cable was lagged around served as ground electrode. Throughout the implantation process, impedance was regularly measured.

### Programming

The chip of the microstimulator is programmed before being mounted onto the board. Programming is done in the socket connected to a computer interface.

### Operating Principle

**FIGURE 2:**
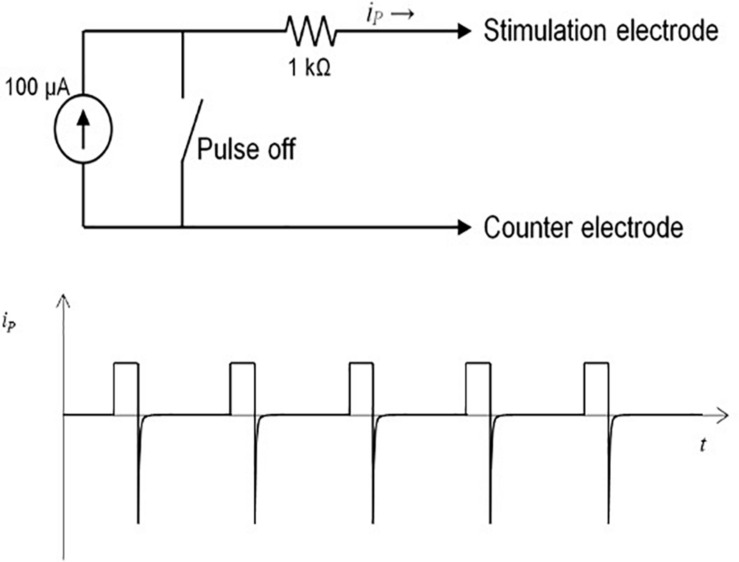
**(A)** Operational principle with 100 μA power and reed switch. **(B)** Monophasic pulses in current over time diagram. After end of pulse current dissipates in the tissue so net charge is zero.

The microstimulator, operated by an 8 bit-microcontroller (C8051F330, Silicon Laboratories), provides monophasic pulses with a pulse width between 60 and 500 μs, an amplitude up to 300 μA, and a frequency 10–500 Hz. All parameters can be programmed individually before implantation. During pulse duration, the current source is set to a constant value, applying charge into the tissue. Between the pulses, the current source is turned off, with a voltage value of zero at the controller output (Figure 2A). This allows the accumulated current to discharge over the 1 kΩ resistance during the pulse off time. Thus, similar to a biphasic pulse, the time-averaged current is set to zero, ensuring no charge accumulation in the tissue (Figure 2B). The maximum battery running time is around 30 days *in vitro* with a 1 kO consumer. A reed contact enables the researcher to switch the microstimulator on and off while being implanted. The current running mode can be observed by a white high-power LED which can be seen shining through the fur. Flash code of the LED is listed in Table 1. As current source serves two commercially available 1.5 V batteries (VARTA V 364, Varta consumer batteries GmbH & Co. KGaA, Ellwangen, Germany). Specifications are summarized in Table 2.

**TABLE 1:** Flash-code of the white LED on the circuit board.

Function	LED
Power off	off
Switch on	5 slow flashes
Running	1 flash roughly each 5 s
Switch off	5 fast flashes
Disconnection in current circle	2 fast flashes

**TABLE 2:** Technical specification of the stimulator.

Specifications	
Battery life	30 days
Batteries	V 364 (Varta)
Supply voltage	3 V
Consumption	∼20 μA
Programmable stimulus	0–500 μA
Frequency range	10–200 Hz
Pulse-width	60–500 μs
Channels	1
Pulse pattern	Monophasic
Control	On/off with switch
Monitoring	On/Off detection with LED diode
Weight (with Batteries)	2.8 g
Stimulator dimensions	9 × 9 × 1 mm

### Animals

In this study, 6–8 weeks old BABLc-Mice were used. All mice were housed under controlled conditions. All animal experiments were approved by the Animal Experimentation Committee of University of Wuerzburg and were carried out in agreement with German laws and European regulations.

### Surgery

Mice were deeply anesthetized using inhalation with 1–2% isoflurane in oxygen and placed in a stereotaxic apparatus (Stoelting, Dublin 6). Body temperature control was achieved by a heating mat. Lidocaine was used as a local anesthetic. During the operation eye ointment (Bepanthen^®^, Dexpanthenol, Bayer Vital GmbH, 51368 Leverkusen) was used to prevent damage to the eyes. The stimulating electrode wire, bare electrode diameter 50 μm, isolated electrode diameter 100 μm was implanted in the right nucleus accumbens core, with the coordinates of anterior-posterior, +1.50 mm; mediolateral, +1.00; dorsoventral −4.50 (all from bregma) at an angle of 0°, based on Allen Mouse Brain Connectivity Atlas (2013). The complete setup was covered in dental cement (Paladur, Heraeus Kulzer GmbH, Hanau) and the rims were trimmed to avoid any sharp edges. After the procedure, the fur was closed completely with interrupted sutures.

**FIGURE 3:**
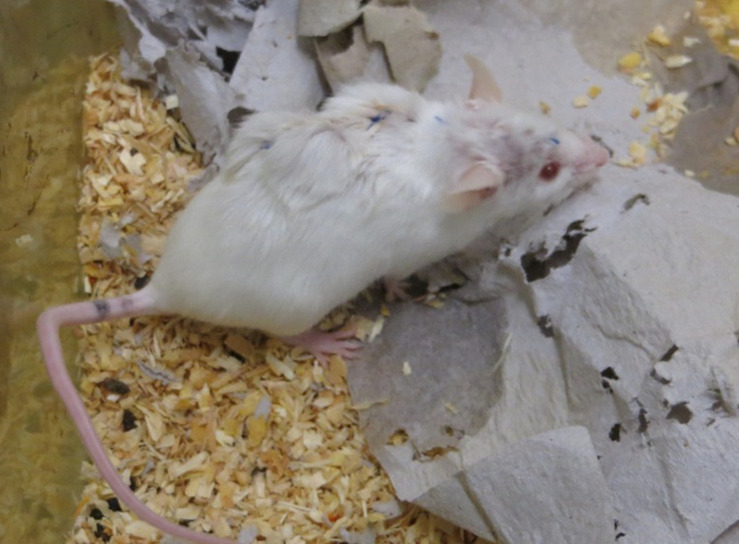
Freely moving mouse with implanted stimulator, ½ h after operation.

The microstimulator was implanted subcutaneously in a reservoir in the back of the mouse attained by traumatically separating the skin from muscle fascia. The connective wires from the electrode and ground screw were tunneled below the skin of the neck from the dorsal cut toward the stimulating device. After connection and insulation of the connector, the fur was closed over the simulation device with interrupted sutures (Figure 3).

All mice received post-operative every 12 h up to 72 h Tramadol subcutaneously (2 mg/kg BW) for pain reduction, antibiosis with Ampicillin (50 mg/kg BW), and 500 μl isotonic saline-chloride as fluid replacement. The mice were able to recover from the operation within 1 week with food and water available *ad libitum*.

### Stimulation

Mice were stimulated for 4 h to provide sufficient time for the expression of c-fos (Herrera and Robertson, 1996; Schulte et al., 2006) and then killed by CO_2_ overdose and fixed with transcardial perfusion of 15 ml phosphate-buffered saline (PBS) and 15 ml body warm 4% paraformaldehyde (PFA). Electrodes were removed, and brains were dissected and kept for post-fixation in 4% PFA for 2 h at room temperature.

### Immunohistochemistry

Coronal brain slices (30 μm) were obtained and free-floating sections were kept in PBS, 4°C. For immunostaining, the slices were blocked in blocking solution of PBS with 10% horse serum, 0.1% Tween 20%, and 0.5% Triton X100 for 2 h and subsequently incubated with primary antibodies at 4°C overnight. The following primary antibodies were used: rabbit polyclonal anti-c-fos antibody (1:10,000, Synaptic System mbH, 37079 Göttingen, Germany) and guinea pig polyclonal anti-NeuN antibody (1/100, Fox3; Synaptic System mbH, 37079 Göttingen, Germany) as the marker of neuronal cells. After rinsing the slices three times with washing solution containing PBS, 0.1% Tween 20%, and 0.1 Triton X100, slices were incubated with secondary antibodies in blocking solution for 2 h. As secondary antibodies were used: Cy3-donkey anti-rabbit antibody (1:800, Jackson ImmunoResearch Laboratories, Inc., West Grove, PA, United States, 19390) and Cy5 donkey anti-guinea pig (1:800, Jackson ImmunoResearch Laboratories, Inc., West Grove, PA, United States, 19390). After incubation, slices were washed three times with washing buffer and counterstained with DAPI (1:5000) for 5 min and afterward rinsed in PBS and mounted on coverslips and covered with aquapolymount (Polysciences Europe GmbH, D-69214 Eppelheim, Germany). Imaging was done on a fluorescence Microscope (Kyence, BZ-8000, KEYENCE Deutschland GmbH, D-63263 Neu-Isenburg).

### C-Fos Expression and Statistical Analysis

Number of c-fos positive cells in the nucleus accumbens shell was determined and ratio to the area was calculated to obtain number of cells/μm^2^. *N* = 4 mice were used for the analysis. Differences in c-fos positive cells/μm^2^ of the stimulated vs. unstimulated side was analyzed with Mann-Whitney-*U* test for nonparametric distribution of values, using GraphPad Version 7 (GraphPad Software, La Jolla California, United States). *p* > 0.05 was considered statistically significant with a confidence interval of 95%.

## Results

For implantation in mice, the circuit board with the controller chip was coated completely (Figure 1c). The batteries were coated separately and connected to the device. Impedance and function of the electrodes and the circuit were tested before. After implantation, the control of the device with the reed contact was tested and running status of the stimulator could be easily controlled by the flash-code of the white LED visible through the fur.

**FIGURE 4:**
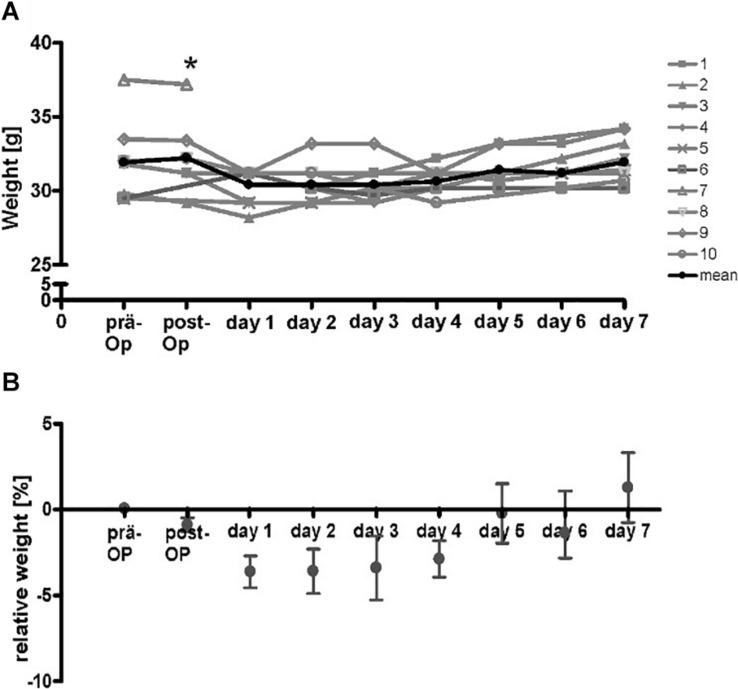
Course of weight of wt-mice (*n* = 10) at the time-points before operation, directly after operation and on the following 7 days. The additional gain of weight of the implanted stimulator was subtracted from values after implantation **(A)** absolute weight-course. * Drop-out. Gray lines, respective mice, black line mean of absolute weight. **(B)** Calculated means of relative weight. Initial weight was reached after 5 days.

To test the compatibility of the implanted device, a small series of 10 animals were implanted and monitored over 7 days. After the operation, maximal loss of weight in the animals was 3.5% of initial weight which is generally accepted as criteria for a not stressful procedure. All animals regained initial weight after 5 days (Figure 4). The mice showed no signs of stress and were in good clinical condition.

**FIGURE 5:**
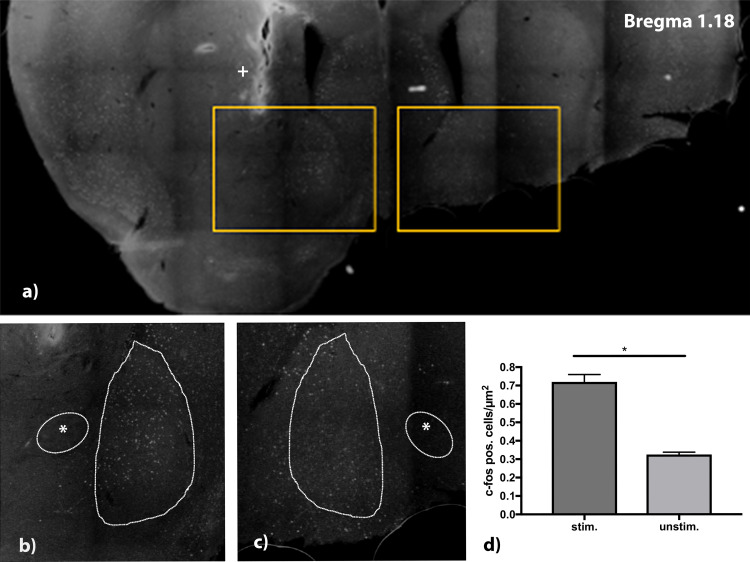
**(a)** C-fos stained brain slice at Bregma 1.18 mm. Electrode was implanted on the right side aiming at the medial shell of nucleus accumbens. Detail view **(b,c)** show upregulation of c-fos in the stimulated side **(b)** vs. lesser expression of c-fos in the unstimulated side. **(d)** Analysis of stimulated (stim.) vs. unstimulated (unstim.) site with a higher number of c-fos positive cells/μm^2^. *n* = 4 animals, mean number of cells/μm2 ± SEM. *Commissura anterior, **+** Electrode artifact.

As a parameter for successful stimulation, expression of the immediate early gene c-fos was tested. The electrode was positioned in the right core region of nucleus accumbens. The position was controlled visually after histological refurbishment and comparison to the Alan mouse brain atlas and showed a correct placement of the uninsulated tip within the core of nucleus accumbens. After stimulation of the mice for 4 h, with stimulation parameters set at 130 Hz, 100 μA, and a pulse width of 60 μs, a higher expression of c-fos in the right shell of nucleus accumbens compared to the non-stimulated contralateral site was found (Figure 5). For quantifications all c-fos positive cells in the area of the nucleus accumbens shell region were counted in a 30 μm thick slice.

## Discussion

Animal models play an essential role in the analysis of the cellular and functional basis of chronically applied high-frequency pulses as used in DBS to specific areas of the brain. Mice are especially useful for this purpose, allowing the analysis of genetically modified animals, thus resembling genetic defects that underlie familial forms of many neuropsychiatric diseases. Standardized behavioral tests have been developed with mice to investigate higher brain functions including attention, learning, memory formation, and retrieval which are relevant for the development of new therapeutic strategies, in particular for psychiatric diseases but also for assessing side effects of DBS which is commonly used for the treatment of movement disorders, in particular Parkinson’s disease. Chronic stimulation in animal models is associated with particular difficulties. The value of behavioral testing data is based on undisturbed movement and behavior of the tested animals. They should be as unhindered by the experimental conditions and devices as possible. Current solutions for devices are either cable-bound or restrict free movement or influence behavior of the animals in other ways (Nowak et al., 2011; de Haas et al., 2012). To overcome the disadvantages of cable-bound stimulation and external parts of the electrodes, we developed a small, fully implantable stimulation device for the stimulation in mice. It was well-tolerated, and the animals gained their initial weight at 5 days after the surgical intervention.

The electrical parameters used in the experimental brain stimulation in mice are monophasic or biphasic rectangular pulses with a pulse width of 60 μs, a current power of 300 μA, and a frequency of 130 Hz (Nowak et al., 2011). We choose a microcontroller operating the energy source in a constant current, voltage-controlled mode, giving off monophasic rectangular pulses. Between the pulses, the voltage is set to zero, allowing the current to dissipate over the internal resistance. Thus, no charge is accumulated in the tissue, and a charged balanced stimulation is achieved, reducing the risk of tissue damage. By turning the current source off between the pulses, the lifespan of the batteries can be extended to provide long-term stimulation. In the test, with a 1 kO resistance to simulate the conditions in the tissue, the stimulator could be used up to 30 days of continuous stimulation. Monopolar pulses were chosen, as the monopolar stimulation leads to less tissue damage as bipolar pulses due to the application of less current in the target region (Temel et al., 2004). Tests in patients have revealed that with constant current modus stimulation, there is no disadvantage of the effect of stimulation in comparison to constant voltage modus (Ramirez de Noriega et al., 2015). Thus, this device resembles conditions that are of clinical relevance and are used for DBS treatment in patients with neuropsychiatric disorders.

To investigate the effect of the current in the target area on a cellular basis, we investigated neuronal c-fos expression. C-fos is a member of the family of the “immediate early genes” comprising c-Fos, FosB, Fra1, and Fra2 (Curran and Morgan, 1995). The expression of these genes is increased in neurons by increased electrical activity. This effect is mediated via the calmodulin kinase pathway with CaMKIV and mitogen-activated protein pathway (MAPK) leading to the phosphorylation of the nuclear transcription factor CREB (cAMP-responsive element-binding protein) (Wu et al., 2001) and increased translation of fos-family genes. Maximum expression of c-fos is expected after 2–4 h upon electrical stimulation (Herrera and Robertson, 1996; Schulte et al., 2006). The detection of increased immediate early gene transcripts are a well-established and verified method to assess neuronal reactions to DBS and have been used previously to monitor neuronal activation in animal models treated by DBS (Klein et al., 2011; Saryyeva et al., 2011).

DBS in the context of complex psychiatric disorders is evolving as a possible therapeutic approach, and basic research in this area is needed. To elicit underlying mechanism new methods of stimulation in mouse models are required that are compatible with extensive behavioral testing. We have chosen the nucleus accumbens as a clinically relevant area for stimulation in addiction disorders (Muller et al., 2013) or depression (Schlaepfer and Bewernick, 2013). The anatomical architecture of the nucleus accumbens can be separated on a histochemical level into a central core and the shell area, which is subdivided into a lateral, medial, dorsomedial, and ventromedial part (Groenewegen et al., 1999). Complex interconnectivity of afferent structures to the nucleus accumbens leads to coordinated network activation of different populations of neurons within the sub-regions of the core (Groenewegen et al., 1999). All of these connections could be targets of DBS effects in this region and thus mediate the clinical effects which have been observed after DBS of this region for treatment of severe alcohol addiction (Muller et al., 2016). We observed a significant increase of c-fos positive neurons in the lateral shell of the nucleus after stimulation of the core region. The connections mentioned above might be mediating the stimulatory effect, but this is a mere observation, and further research is necessary to identify the actual structure or fibers leading to the activation of the lateral shell neurons.

With the development of a fully implantable stimulator for mice, an urgent need is met for further research on the effects of deep brain stimulation. It offers the possibility to conduct behavioral experiments in freely moving mice for up to 30 days of stimulation. The stimulating effect was proven with the increase of c-fos in the stimulated area after preimplantation in mice.

## Data Availability Statement

All datasets generated for this study are included in the article/supplementary material, further inquiries can be directed to the corresponding authors.

## Ethics Statement

The animal study was reviewed and approved by the Animal Experimentation Committee of the University of Wuerzburg.

## Author Contributions

MF, HE, and MS: experimental concept and design and drafting of the manuscript. MF: experimental procedures, animal handling and analysis, and statistical analysis. MS and JV: critical revision of the manuscript for important intellectual content. HE, MS, and JV: experimental supervision. All authors had full access to all of the study data and took responsibility for the integrity and accuracy of the data analysis.

## Conflict of Interest

The authors declare that the research was conducted in the absence of any commercial or financial relationships that could be construed as a potential conflict of interest.
